# Bronchoalveolar lavage fluid reveals factors contributing to the efficacy of PD-1 blockade in lung cancer

**DOI:** 10.1172/jci.insight.157915

**Published:** 2022-05-09

**Authors:** Kentaro Masuhiro, Motohiro Tamiya, Kosuke Fujimoto, Shohei Koyama, Yujiro Naito, Akio Osa, Takashi Hirai, Hidekazu Suzuki, Norio Okamoto, Takayuki Shiroyama, Kazumi Nishino, Yuichi Adachi, Takuro Nii, Yumi Kinugasa-Katayama, Akiko Kajihara, Takayoshi Morita, Seiya Imoto, Satoshi Uematsu, Takuma Irie, Daisuke Okuzaki, Taiki Aoshi, Yoshito Takeda, Toru Kumagai, Tomonori Hirashima, Atsushi Kumanogoh

**Affiliations:** 1 Department of Respiratory Medicine and Clinical Immunology, Osaka University Graduate School of Medicine, Osaka, Japan.; 2 Department of Immunopathology, World Premier International Research Center, Immunology Frontier Research Center, Osaka University, Osaka, Japan.; 3 Department of Thoracic Oncology, Osaka International Cancer Institute, Osaka, Japan.; 4 Department of Immunology and Genomics, Graduate School of Medicine, Osaka City University, Osaka, Japan.; 5 Division of Metagenome Medicine, Human Genome Center, The Institute of Medical Science, The University of Tokyo, Tokyo, Japan.; 6 Division of Cancer Immunology, Research Institute/Exploratory Oncology Research and Clinical Trial Center, National Cancer Center, Tokyo, Japan.; 7 Department of Otorhinolaryngology-Head and Neck Surgery, Osaka University Graduate School of Medicine, Osaka, Japan.; 8 Department of Thoracic Oncology, Osaka Habikino Medical Center, Osaka, Japan.; 9 Department of Cellular Immunology, Research Institute for Microbial Diseases, Osaka University, Osaka, Japan.; 10 Division of Health Medical Intelligence, Human Genome Center, The Institute of Medical Science, The University of Tokyo, Tokyo, Japan.; 11 Genome Information Research Center, Research Institute for Microbial Diseases, Osaka University, Osaka, Japan.; 12 Integrated Frontier Research for Medical Science Division, Institute for Open and Transdisciplinary Research Initiatives, and; 13 Center for Infectious Diseases for Education and Research, Osaka University, Osaka, Japan.

**Keywords:** Immunology, Oncology, Cancer immunotherapy, Chemokines, Lung cancer

## Abstract

Bronchoalveolar lavage is commonly performed to assess inflammation and identify responsible pathogens in lung diseases. Findings from bronchoalveolar lavage might be used to evaluate the immune profile of the lung tumor microenvironment (TME). To investigate whether bronchoalveolar lavage fluid (BALF) analysis can help identify patients with non–small cell lung cancer (NSCLC) who respond to immune checkpoint inhibitors (ICIs), BALF and blood were prospectively collected before initiating nivolumab. The secreted molecules, microbiome, and cellular profiles based on BALF and blood analysis of 12 patients were compared with regard to therapeutic effect. Compared with ICI nonresponders, responders showed significantly higher CXCL9 levels and a greater diversity of the lung microbiome profile in BALF, along with a greater frequency of the CD56^+^ subset in blood T cells, whereas no significant difference in PD-L1 expression was found in tumor cells. Antibiotic treatment in a preclinical lung cancer model significantly decreased CXCL9 in the lung TME, resulting in reduced sensitivity to anti–PD-1 antibody, which was reversed by CXCL9 induction in tumor cells. Thus, CXCL9 might be associated with the lung TME microbiome, and the balance of CXCL9 and lung TME microbiome could contribute to nivolumab sensitivity in patients with NSCLC. BALF analysis can help predict the efficacy of ICIs when performed along with currently approved examinations.

## Introduction

Immune checkpoint inhibitors (ICIs) have drastically changed the therapeutic strategies for advanced non–small cell lung cancer (NSCLC). Only 20% of all patients with NSCLC benefit from monotherapy with the anti–PD-1 antibody nivolumab as second- or later-line treatment (1). PD-L1 expression on NSCLC cells is a clinically established biomarker that helps determine whether to use PD-1 blockade monotherapy or combination therapy with cytotoxic reagents according to the patient condition. However, this biomarker is susceptible to tumor heterogeneity, and it does not adequately predict the efficacy of PD-1/PD-L1 blocking treatment in other malignancies (2). Therefore, additional biomarkers that can be collected by noninvasive examination in daily practice are necessary to improve predictive accuracy. From this perspective, analyzing bronchoalveolar lavage fluid (BALF) can be a promising strategy, especially in NSCLC, because bronchoscopy is an established clinical procedure to evaluate the local immune status in lung disease, and it is generally performed to diagnose lung cancer. BALF is a relatively noninvasive method, which might be beneficial for assessing immune profiles in the lung tumor microenvironment (TME) and for predicting the efficacy of ICIs.

Growing evidence regarding the interactions between the host and its microbiome has revealed potential implications for this relationship in health and various diseases, including cancer (3). While the effects of local and systemic immunological modulation by the gut microbiome have been intensively investigated, those of the lung microbiome have not been thoroughly explored, because the organisms that constitute it were only recently accepted as an important part of the resident microbial population (4). Nevertheless, a number of studies have suggested that the lung microbiome plays an important role in the development of lung diseases, including lung cancer (5–7). A recent report demonstrated that the lung immunological signature seems to be more closely correlated to the lung microbiome than to the gut microbiome (8), although the number of organisms is much smaller in the respiratory tract than in the gut. Several studies have shown that the gut microbiome signature is associated with sensitivity to ICIs (9–12), and that antibiotic (ABx) treatment prior to ICI initiation reduces the efficacy of ICIs in patients with cancer (13). However, it remains unknown how the airway microbiome modulates the sensitivity to ICIs in patients with NSCLC.

In this prospective cohort study, we collected BALF and PBMCs before initiating nivolumab treatment in patients with NSCLC. We found that, compared with ICI nonresponders, responders showed significantly higher CXCL9 levels and greater diversity in the lung microbiome profile in BALF supernatant, as well as an enriched transcriptome profile of cytotoxic T cell activation in BALF cells. In addition, CD56^+^CD4^+^ T cells and CD56^+^CD8^+^ T cells in PBMCs were increased in responders compared with nonresponders. A preclinical murine lung cancer model using intrathoracic injection (14, 15) of the *Kras*-mutated, *Tp53*-deficient lung cancer cell line KP9-3 (16) demonstrated that ABx treatment reduced CXCL9 production in the lung TME and hampered CXCR3^+^CD8^+^ T cell infiltration induced by anti–PD-1 blocking antibodies, leading to reduced efficacy of anti–PD-1–blocking treatment. Thus, the lung microbiome could be linked to CXCL9-dependent CD8^+^ T cell recruitment in the lung TME, which potentially modulates the sensitivity to ICIs.

## Results

### Patient characteristics.

Twenty-four patients with NSCLC were enrolled in this study (Table 1 and Supplemental Figure 1; supplemental material available online with this article; https://doi.org/10.1172/jci.insight.157915DS1). Ten patients were excluded because they harbored driver mutations, including *EGFR* and *ALK* mutations, that are correlated with a low response rate to anti–PD-1 antibodies (17, 18). In addition, two patients were excluded because their target lesions could not be evaluated. Eventually, blood and BALF samples from 12 patients were obtained before initiating nivolumab monotherapy (Supplemental Figure 2A). The objective response was evaluated by RECIST criteria (version 1.1) at 6 weeks after treatment and was repeated every 6 weeks thereafter. Six patients whose target lesions showed partial response or stable disease were defined as responders, and the other 6 patients whose target lesions showed progressive disease were defined as nonresponders. Clinical outcomes for all patients are shown in a waterfall plot and swimmer plot (Supplemental Figure 3, A and B). There was no significant difference in patient characteristics between responders and nonresponders (Table 1).

### CXCL9 in BALF and CD56^+^ peripheral T cells were significantly elevated in responders compared with nonresponders.

**Figure 1:**
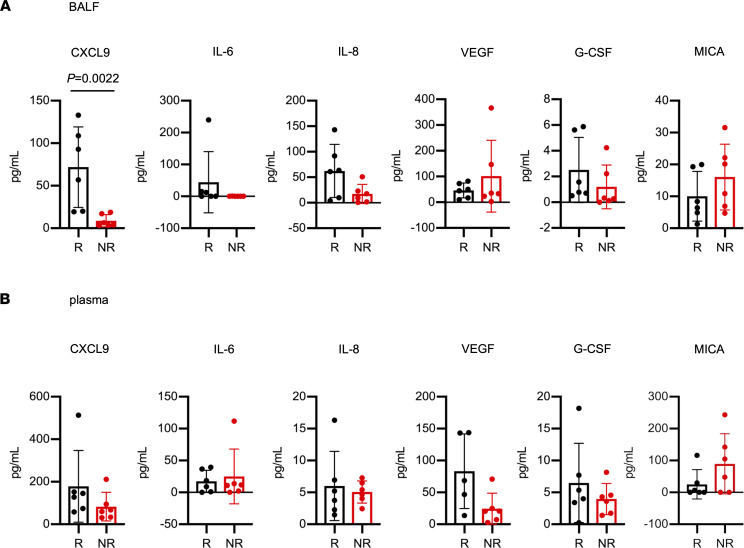
CXCL9 levels in BALF were significantly elevated in responders compared with nonresponders. Comparison of cytokine levels in (**A**) BALF and (**B**) plasma from responders (R; black) and nonresponders (NR; red) before initial nivolumab treatment. Cytokine levels were measured by ELISA and CBA. Data are presented as the mean ± SEM. *P* values were calculated using the Mann-Whitney *U* test.

Thirteen factors secreted in BALF and plasma were selected from previous publications (16, 19–25) and were measured (Figure 1 and Supplemental Figure 4, A and B). No significant differences in any factors were found in plasma between responders and nonresponders, while in BALF only CXCL9 was significantly elevated in responders compared with nonresponders (*P* = 0.0022). Higher CXCL9 levels in BALF were correlated with significantly prolonged progression-free survival (Supplemental Figure 3C). Because CXCL9 levels in BALF were significantly higher in responders, we also measured the levels of CXCL10, which is a major ligand for CXCR3 and, like CXCL9, is essential for T cell recruitment. We assessed CXCL10 levels using residual BALF samples after another freeze–thaw process and demonstrated higher levels in the responders, although the difference was not significant (*P* = 0.063) (Supplemental Figure 4C).

Mass cytometry analysis of 32 markers was performed for immune cell profiling of PBMCs (details listed in Supplemental Table 1). The frequencies of the CD56^+^ and CD69^+^ subsets of CD4^+^ T cells and the CD56^+^ subset of CD8^+^ T cells were significantly increased in responders compared with nonresponders (Supplemental Figure 5). Seven BALF cell samples were successfully analyzed by bulk RNA sequencing: 4 samples from responders and 3 from nonresponders. Of 115 genes (normalized counts > 10) that were significantly (*P* < 0.05) and differentially (log_2_ fold change > 2) expressed between responders and nonresponders, 87 were upregulated and 28 were downregulated in responders compared with nonresponders (Supplemental Figure 6 and Supplemental Tables 2 and 3). The expression of T cell markers (*CD3E*, *CD3G*, and *CD8A*), activation markers of cytotoxic T cells (*GZMB* and *IL2RB*), and *CXCR3*, a receptor for CXCL9, was clearly elevated in BALF cells from responders. On the other hand, genes related to immunosuppression, such as *LAMA1* (26) and *CARM1* (27), were enriched in nonresponders.

### Microbiome diversity in BALF was greater in nonresponders compared with responders.

**Figure 2:**
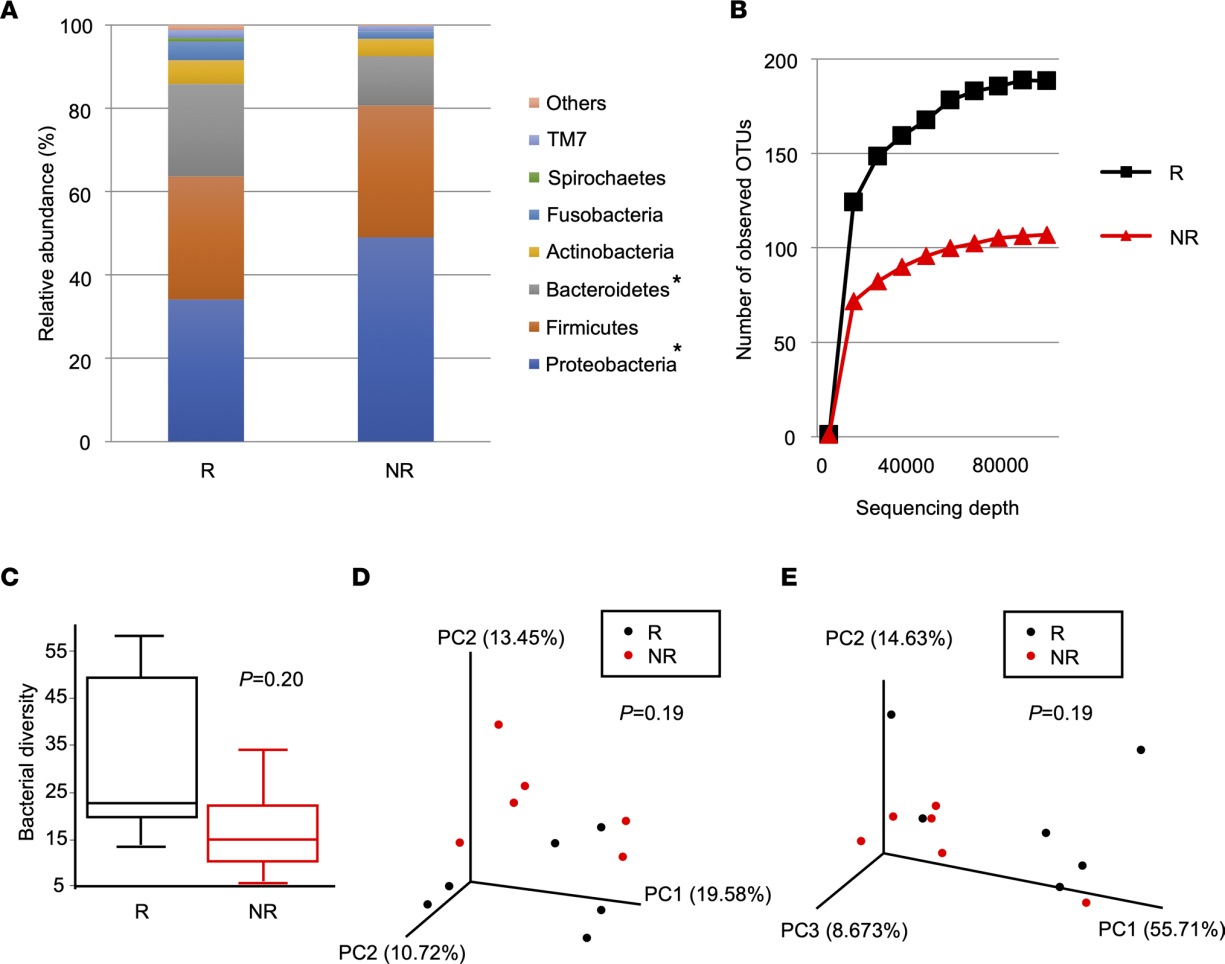
Respiratory microbial diversity was reduced in nonresponders compared with responders. (**A**) Respiratory microbial composition at the phylum level based on the relative abundance of operational taxonomic units (OTUs) for the BALF samples. *P* values were calculated using unpaired *t* test. *P < 0.05. (**B** and **C**) Alpha diversity analysis of the respiratory microbiome. (**B**) Rarefaction curve using the number of OTUs. (**C**) Bacterial alpha diversity based on Faith’s phylogenetic alpha diversity index. *P* values were calculated using the Kruskal-Wallis test. (**D** and **E**) Principal coordinate analysis of (**D**) the unweighted UniFrac distance matrices and (**E**) the weighted UniFrac distance matrices for the microbial communities. *P* values were calculated using the permutational multivariate ANOVA test. Responders (R; black), *n* = 6. Nonresponders (NR; red), *n* = 6.

To investigate how respiratory microbial profiles differ between responders and nonresponders, we performed 16S ribosomal RNA gene sequencing of BALF supernatants. The respiratory microbial compositions at the phylum level showed that the relative abundance of Proteobacteria was significantly lower, whereas that of Bacteroidetes was significantly higher, in responders compared with nonresponders (Figure 2A and Supplemental Figure 7A). Bacterial diversity evaluation within each sample showed that bacterial alpha diversity was higher in responders compared with nonresponders (Figure 2, B and C). Comparison of β diversities in BALF between responders and nonresponders revealed no differences in unweighted UniFrac (Figure 2D) or weighted UniFrac (Figure 2E). Thus, altered microbial compositions and reduced bacterial diversity, which indicate dysbiosis, were confirmed in nonresponders.

### Dysbiosis was associated with decreased efficacy of a PD-1 inhibitor through reduction of CXCL9 levels in TME.

**Figure 3:**
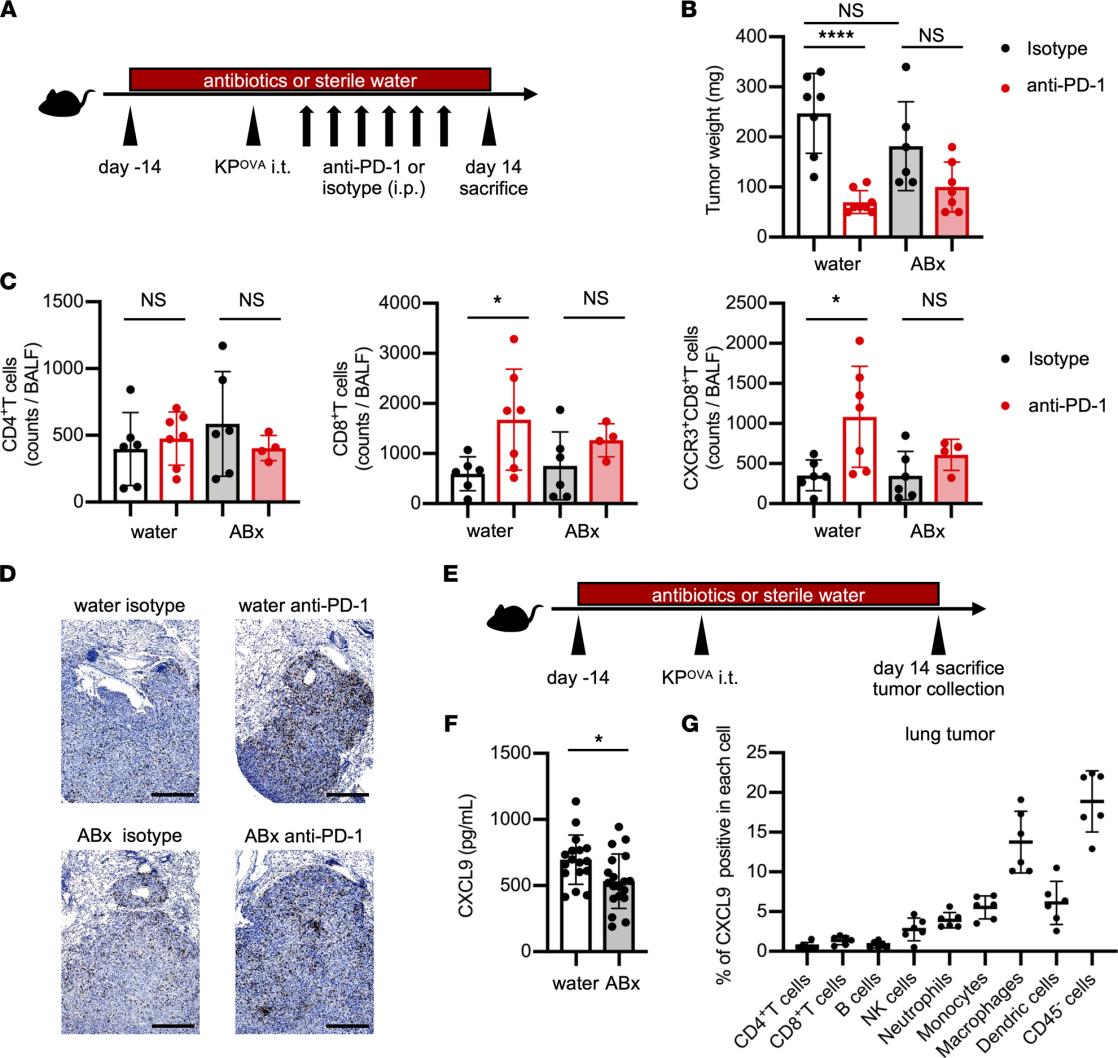
Dysbiosis suppressed both CXCL9 secretion in the tumor microenvironment and CD8^+^ T cell recruitment, leading to attenuated efficacy of PD-1 blockade. (**A**) Schematic of treatment schedule. Before tumor inoculation, mice were pretreated with an ABx cocktail of ampicillin, neomycin, vancomycin, and metronidazole or with sterile water. After 2 weeks, mice were inoculated intrathoracically (i.t.) with 1 × 10^6^ KP^OVA^ cells and then treated i.p. with 200 μg of either anti–PD-1 antibodies or isotype control for 2 weeks (3 times/week) starting 3 days after tumor inoculation. BALF and lung tumors were collected after sacrifice on day 14. (**B**) Tumor weight of mice that received sterile water or ABx that were then treated with isotype (black) or anti–PD-1 (red). Tumor weight encompasses the total weight of the tumor enucleated from the lung tissue and tumors invading the mediastinum and chest wall (*n* = 6–7 mice/group). (**C**) BALF was collected by washing mouse lungs with 1 mL PBS. The number of each T cell subset was analyzed by flow cytometry (*n* = 4–7 mice/group). (**D**) Images of immunohistochemistry staining of CD8 (brown) in tumor tissue of each representative sample. Original magnification, × 4. Scale bar: 500 μm. (**E**) Schematic of treatment schedule. Mice were pretreated with ABx or sterile water for 2 weeks before KP^OVA^ inoculation. BALF and lung tumors were collected after sacrifice on day 14. Lung tumors were enucleated and homogenized for measurement of CXCL9. (**F**) Concentrations of CXCL9 in supernatants of homogenized lung tumors (white bar, sterile water group; gray bar, ABx group) were measured by ELISA (*n* = 17–19 mice/group). (**G**) Percentage of CXCL9^+^ cells among tumor-infiltrating leukocytes (CD45^+^) and cancer cells (CD45^-^) was analyzed by flow cytometry after stimulation with IFN-γ and lipopolysaccharide. The graph represents each lung tumor nodule (*n* = 6). (**B**, **C**, **F**, and **G**) Data are representative of at least 2 independent experiments. Data are presented as the mean ± SEM. *P < 0.05; statistical significance determined by Student’s *t* test. ****P < 0.0001; statistical significance determined by 1-way ANOVA with Tukey’s multiple comparison test.

The results obtained in this clinical study suggested that impaired CXCL9 production and dysbiosis in BALF might also be present in the lung TME of patients with NSCLC, which could reduce the sensitivity to PD-1–blocking treatment. To investigate this, we developed a murine lung cancer model (Supplemental Figure 8A) and evaluated whether dysbiosis reduced the CXCL9 level in BALF and the antitumor effect of anti–PD-1 antibodies in vivo. C57BL/6J mice were treated with ABx or sterile water for 2 weeks before tumor injection, and then 1 × 10^6^ OVA-expressing, *Kras*-mutated, *Tp53*-deficient lung adenocarcinoma (KP^OVA^) cells were inoculated intrathoracically. After tumor inoculation, the ABx and the sterile water (control) groups were each subdivided into 2 groups: an anti–PD-1 antibody–treated group and an isotype control-treated group (Figure 3A). In the control group, tumor growth was significantly reduced in mice treated with anti–PD-1 antibody compared with those treated with isotype control. By contrast, no significant difference was found in the ABx group (Figure 3B). We collected BALF from mice on day 14 to assess immune cell profiles of the TME. In the control group, CD8^+^ T cells, in particular CXCR3^+^CD8^+^ T cells, were significantly increased by anti–PD-1 antibody compared with isotype control, but no differences were found in the ABx group (Figure 3, C and D). These results suggest that the lung microbiome may influence the efficacy of anti–PD-1 treatment by modulating the recruitment of activated CD8^+^ T cells to lung tumors.

We then investigated whether dysbiosis affected CXCL9 secretion in the TME, as shown in the clinical study. Mice were treated with ABx or sterile water (control) for 2 weeks before intrathoracic KP^OVA^ cell injection and sacrificed 2 weeks after injection (Figure 3E). Lung tumors were isolated and CXCL9 in the supernatant of homogenized tumor cells was measured. The CXCL9 levels were significantly higher in lung tumors in the control group than those in the ABx group (Figure 3F), suggesting that dysbiosis can affect the secretion of CXCL9 in the lung TME. Given these results, we analyzed the correlation between CXCL9 levels and bacterial diversity in BALF from patients. We found that CXCL9 levels showed a significant positive correlation with bacterial diversity (*P* = 0.049, *r* = 0.59) (Supplemental Figure 7B). To determine what cells were the main source of CXCL9 in the lung TME, CXCL9 expression in each lung tumor cell population was evaluated by flow cytometry. CXCL9 positivity was mainly identified in cancer cells and myeloid cells, especially macrophages and dendritic cells (Figure 3G and Supplemental Figure 8B).

### Overexpression of CXCL9 in KP^OVA^ cells enhanced recruitment of tumor-specific CD8^+^ T cells and reduced tumor growth.

**Figure 4:**
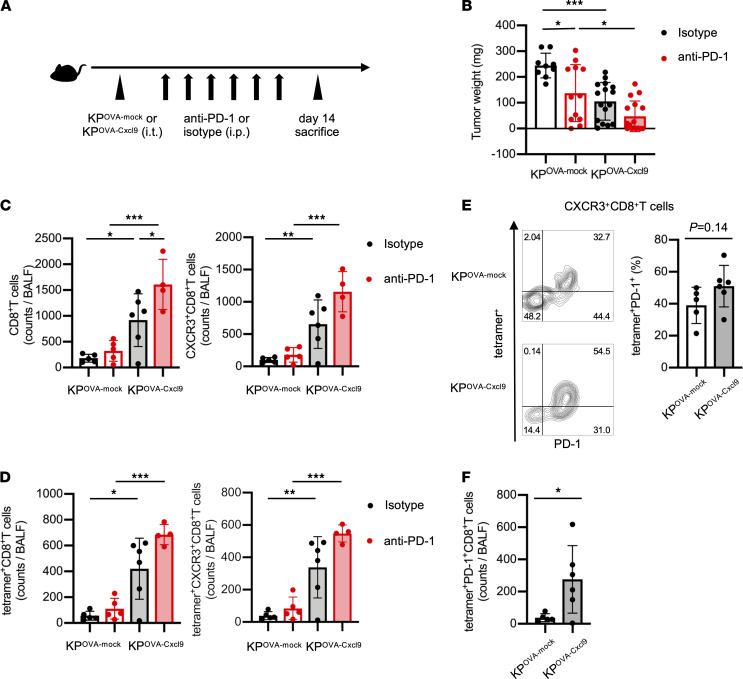
Overexpression of CXCL9 in KP^OVA^ enhanced recruitment of tumor-specific CXCR3^+^CD8^+^ T cells and reduced tumor growth. (**A**) Schematic of treatment schedule. Mice were inoculated with 1 × 10^6^ KP^OVA^ cells transfected with empty vector (mock) or with KP^OVA-Cxcl9^ cells. Then, they were treated with either anti–PD-1 antibodies or isotype control for 2 weeks starting 3 days after tumor inoculation. BALF and tumors were collected after sacrifice on day 14. (**B**) Tumor weight of mice inoculated with KP^OVA-mock^ or KP^OVA-Cxcl9^ cells and treated with isotype (black) or anti–PD-1 (red). Tumor weight encompasses the total weight of the tumor enucleated from the lung tissue and tumors invading the mediastinum and chest wall. Data are presented as the mean ± SEM. *P < 0.05, *** *P* < 0.001; statistical significance determined by 1-way ANOVA with Tukey’s multiple comparison test (*n* = 9–16 mice/group). (**C** and **D**) Total counts of (**C**) CD8^+^ and CXCR3^+^CD8^+^ T cells and (**D**) tetramer^+^ and tetramer^+^CXCR3^+^CD8^+^ T cells in BALF were analyzed by flow cytometry. BALF was collected from mice inoculated with KP^OVA-mock^ or KP^OVA-Cxcl9^ cells and treated with isotype (black) or anti–PD-1 (red). Data are presented as the mean ± SEM. *P < 0.05, ** *P* < 0.01, ***P < 0.001; statistical significance determined by 1-way ANOVA with Tukey’s multiple comparison test (*n* = 4–6 mice/group). (**E**) Representative contour plots and summary of the frequency of OVA-tetramer^+^PD-1^+^ subsets in CXCR3^+^CD8^+^ T cells compared between mice inoculated with KP^OVA-mock^ or KP^OVA-Cxcl9^ cells and treated with isotype control. Data are presented as the mean ± SEM. Statistical significance determined by Student’s *t* test (*n* = 5–6 mice/group). (**F**) Summary of the total counts of OVA-tetramer^+^PD-1^+^CD8^+^ T cells in BALF compared between mice inoculated with KP^OVA-mock^ or KP^OVA-Cxcl9^ and treated with isotype control. Data are presented as the mean ± SEM. *P < 0.05; statistical significance determined by Student’s *t* test (*n* = 5–6 mice/group). (**B–F**) Data are representative of at least 2 independent experiments.

CXCL9 secretion in the TME induced T cell infiltration into tumors, leading to improved efficacy of anti–PD-1 treatment. To further analyze this phenomenon, we prepared *Cxcl9*-overexpressing KP^OVA^ (KP^OVA-Cxcl9^) cells (Supplemental Figure 9A). There was no significant difference in either H-2K^b^-SIINFEKL induction by IFN-γ or in cell proliferation between KP^OVA-Cxcl9^ and KP^OVA-mock^ cells (Supplemental Figure 9, B and C). A total of 1 × 10^6^ KP^OVA-Cxcl9^ or KP^OVA-mock^ cells were inoculated intrathoracically into mice, and mice were then treated with anti–PD-1 antibody or isotype control (3 times/week, for 2 weeks) (Figure 4A). As expected, tumor growth in KP^OVA-Cxcl9^-inoculated mice was significantly suppressed relative to that in KP^OVA-mock^-inoculated mice after treatment with either anti–PD-1 antibody or isotype control (Figure 4B). Flow cytometry analysis of BALF cells revealed that not only CD8^+^ T cells and CXCR3^+^CD8^+^ T cells, but also tetramer^+^CD8^+^ T cells and tetramer^+^CXCR3^+^CD8^+^ T cells, were significantly increased in the KP^OVA-Cxcl9^ group compared with the KP^OVA-mock^ group (Figure 4, C and D). Focusing on CXCR3^+^CD8^+^ T cells, the frequencies of tetramer^+^PD-1^+^ cells were elevated in the KP^OVA-Cxcl9^ group compared with the KP^OVA-mock^ group (Figure 4E), and the absolute counts of tetramer^+^PD-1^+^CD8^+^ T cells were significantly increased in the KP^OVA-Cxcl9^ group compared with the KP^OVA-mock^ group (Figure 4F).

## Discussion

This clinical trial sought to determine whether BALF analysis could help identify patients with NSCLC who would respond to ICIs. Bronchoscopy to obtain BALF was performed safely, and potential biomarkers to predict the efficacy of anti–PD-1 antibodies were identified. Investigation of BALF and PBMC samples from ICI responders and nonresponders revealed that BALF with increased CXCL9 levels and greater diversity of the lower respiratory tract microbiome was associated with a clinical response, although the study population was very small and only one baseline BALF sample was evaluated. Intriguingly, PD-L1 expression in tumors in this cohort was unrelated to clinical response, suggesting that data on CXCL9 levels in BALF might compensate for the shortcomings of the PD-L1 staining in patients with NSCLC. CXCL9 interacts with CXCR3, which is highly expressed in CD8^+^ T cells, and recruits T cells into tumors (28–31). CXCL9 is also reportedly involved in Th1 differentiation and activation (32). We found that CXCL9 was mainly produced by epithelial cells, macrophages, and dendritic cells, as reported in other studies (33–35). A recent study of whole-exome and transcriptome data from more than 1000 bulk tumor tissues of 7 cancer types that were treated with ICIs reported that CXCL9 expression was one of the strongest predictors of a therapeutic response (36). However, the present study is the first to our knowledge to confirm that in ICI responders, but not nonresponders, CXCL9 levels were significantly elevated in BALF but not in plasma. These results strongly suggest that CXCL9 in BALF can serve as a vital biomarker that is not affected by tumor tissue heterogeneity and is superior to plasma CXCL9 in terms of sensitivity. The transcriptome profile of BALF cells demonstrated higher *CXCR3* expression in responders compared with nonresponders. By contrast, the expression of immunosuppressive molecules such as *LAMA1* and *CARM1* was elevated in nonresponders compared with responders. A recent study showed that *LAMA1*, a subunit of laminin, was associated with poor prognosis and was negatively correlated with CD8^+^ T cell infiltration in ovarian cancer (26). Another report demonstrated that *CARM1*, an epigenetic enzyme and cotranscriptional activator, attenuated T cell activation and infiltration, and *CARM1* inactivation enhanced the type I interferon response and sensitized resistant tumors to checkpoint blockade (27). These molecules may also be involved in resistance to anti–PD-1 antibody treatment. Further investigation is warranted.

We discovered that, like CXCL9 levels, the diversity of the lower respiratory tract microbiome was reduced in nonresponders compared with responders. The lower respiratory tract was originally considered sterile; however, in recent years, the composition of the bacterial flora in this region has been analyzed in healthy individuals and has been found to be involved in the exacerbation of lung diseases such as COPD and asthma (37–40). A number of lung bacterial studies have also been reported to be associated with lung cancer (41–45). Although many studies have demonstrated that specific bacteria in the gut microbiome are involved in the response to ICIs (9–12, 46), there are few reports on the relation of the lung microbiome to ICI responses. We confirmed that ABx treatment reduced the CXCL9 level in lung tumors in our preclinical model, suggesting that CXCL9 elevation and increased diversity of the lung microbiome in BALF could be linked to each other. However, we used a syngeneic model with a simplified tumor antigen; therefore, the model cannot fully emulate complicated patient lung tumors. Previous studies involving bronchoscopic analysis showed that Gram-negative bacilli, such as *Haemophilus influenzae*, *Enterobacter* sp., *Escherichia coli* (41), and *Firmicutes* and *Saccharibacteria* (42), colonized the respiratory tract of patients with lung cancer. In addition, certain members of the oral microbiome in the lower respiratory tract, such as *Streptococcus, Prevotella*, and *Veillonella*, are associated with poor prognosis in patients with lung cancer (47). These bacteria potentially stimulate pattern recognition receptors, including Toll-like receptors, in bronchial cells, macrophages, and dendritic cells, which leads to inflammatory responses such as the production of Th1-type cytokines and chemokines (48–50). A recent study showed that intratracheal administration of neomycin ameliorated the activity of experimental autoimmune encephalomyelitis (51). In this study, neomycin changed the lung microbiome in rats such that it contained Gram-negative, lipopolysaccharide-enriched phyla, especially Bacteroidetes, which induced type I interferon–primed inflammation. Intriguingly, the neomycin-induced lung microbiome profile was more similar to the profiles of ICI responders compared with nonresponders in our cohort, suggesting that specific bacterial strains classified in Bacteroidetes in the lung microbiome could contribute to increased ICI sensitivity through the type I interferon immune response. Unfortunately, we could not identify the specific bacterial species involved in the therapeutic effect of the ICI in this study. However, as several clinical trials have shown (10, 52–55), certain bacteria might stimulate the innate immune response in ICI responders, leading to CXCL9 production in the TME and sensitization of anti–PD-1 antibody treatment through recruitment of tumor-specific CD8^+^ T cells. Therefore, it will be interesting in future studies to determine which bacterial strains in the respiratory tract are able to induce CXCL9 production. Although relatively few participants were enrolled in this study, the expression of PD-L1 on lung tumor cells at diagnosis was not significantly different between responders and nonresponders, and our results at least suggest that the CXCL9 level in BALF predicts ICI treatment efficacy independently of PD-L1 expression in tumor cells. As in general practice, BALF was obtained in this study without any serious complications. Because bronchoscopy is commonly performed when diagnosing lung cancer, obtaining BALF in addition to biopsy specimens might be a promising option to increase predictive ability. However, this strategy is only applicable to lung cancers and other cancers originating from the respiratory tract, and it is not viable in the context of CT-guided biopsies for diagnosing peripheral target lesions. Furthermore, the results of BALF analyses might identify novel mechanisms to sensitize or desensitize patients to ICI treatment, because BALF is superior to biopsy specimens in a number of ways, especially regarding the analysis of secreted molecules from the lung TME. Validation with a large prospective cohort is required.

## Methods

### Study design and clinical sample collection.

Twenty-four patients with NSCLC who intended to begin nivolumab therapy as second- or later-line treatment at Osaka International Cancer Institute and Osaka Habikino Medical Center between February 2017 and December 2018 were enrolled in this study (Supplemental Figure 1). Ten patients were excluded because they harbored driver mutations such as *EGFR* and *ALK* mutation, and 2 patients were excluded because their target lesions could not be evaluated. Blood and BALF samples were collected from 12 patients before initiating nivolumab treatment. The objective response was evaluated based on the sum of the diameter of target lesions, including primary and metastatic tumors, according to the RECIST criteria (version 1.1) at 6 weeks after initial treatment, and was repeated every 6 weeks thereafter. Eleven patients received initial nivolumab treatment within a few days after BALF collection, and only 1 patient began treatment 1 month later. At the time of initial nivolumab administration, none of the patients received any medication that affected CXCL9 concentrations or bacterial compositions, such as ABx, corticosteroids, and immunosuppressants.

### Processing of BALF and blood.

BAL was performed following the standard protocol, which involved injecting three 50 mL volumes of normal saline (150 mL in total) into the wedged segmental bronchus leading to the target tumor lesion that was assessed radiologically (Supplemental Figure 2, B–E). Blood and BALF were collected in tubes on the same date, and plasma and BALF supernatants were collected after centrifugation and stored at −80°C for analysis. Cells in BALF were stored in RNAlater Stabilization Solution (Thermo Fisher Scientific) for RNA sequencing. PBMCs were isolated using Ficoll-Paque density gradient centrifugation (GE Healthcare) and stored in CELLBANKER 1 (Takara Bio) at −80ºC for analysis.

### Measurement of soluble factors in plasma and BALF supernatant.

IL-6, vascular endothelial growth factor, MHC class I–related protein A, and semaphorin 7A (Sema7A) concentrations were measured by ELISA (Quantikine and Duoset ELISA kits; R&D Systems). BALF samples were undiluted and plasma samples were diluted 1:2. Sema7A concentrations were measured with a Cloud-Clone Corp ELISA Kit. IL-1β, IL-4, IL-8, IL-13, IL-17A, G-CSF, CXCL9, TNF, and IFN-γ concentrations were measured by cytometric bead array (CBA) (BD Biosciences). The standard and samples were applied to 96-well polypropylene Falcon V-bottom plates (Corning), and then capture beads diluent was added and incubation was performed for 1 hour. Next, detection reagent was applied to each well and incubated for 2 hours. After washing, samples were acquired on a flow cytometer (BD FACSCanto II). Analysis was performed using FCAP Array software (BD Biosciences). CXCL10 was measured by CBA using residual BALF samples after an additional freeze–thaw process.

### Bacterial 16S ribosomal RNA–encoding gene sequencing of BALF supernatant.

Bacterial DNA from each BALF sample was extracted using the PowerSoil DNA Isolation Kit (QIAGEN). The 16S ribosomal RNA V3–V4 region was amplified by PCR, and the amplicon was purified as described previously (56). For each sample, equal amounts of each DNA amplicon library were mixed and sequenced on a MiSeq instrument (Illumina) using a MiSeq v3 Reagent kit and a 15% PhiX spike (Illumina). 16S ribosomal RNA gene analysis was performed using QIIME2 (https://qiime2.org). Briefly, raw sequence data were subjected to primer sequence trimming, quality filtering, and paired-end read merging using the dada2 denoise-paired method (–p-trim-left-f 17 –p-trim-left-r 21 –p-trunc-len-f 275 –p-trunc-len-r 215 –p-n-threads 4) (57). Alpha and beta diversity analyses were performed using qiime diversity core-metrics-phylogenetics based on rarefied sample sequences (–p-sampling-depth 84,532 in Figure 2). Before taxonomic analysis, sequences of the 16S ribosomal RNA V3–V4 region were extracted from Greengenes 13_8 99% operational taxonomic units and our primer sequences (forward primer, ACACGACGCTCTTCCGATCTCCTACGGGNGGCWGCAG and reverse primer, GACGTGTGCTCTTCCGATCTGACTACHVGGGTATCTAATCC) (58) using the q2-feature-classifier. Then, the Naive Bayes classifier was trained using the extracted Greengenes 13_8 reference sequences and Greengenes 13_8 99% operational taxonomic unit taxonomy. The taxonomic composition was visualized using a qiime taxa bar plot. Faith’s phylogenetic alpha diversity estimate and principal coordinate analysis of the unweighted and weighted UniFrac distance matrices were performed using QIIME2.

### Mass cytometry analysis using cytometry by TOF.

The antibody panel utilized for cytometry by TOF (CyTOF) is shown in Supplemental Table 1. Some antibodies were labeled with metals using a MAXPAR X8 Polymer labeling kit (Fluidigm). Cisplatin containing isotopically enriched ^194^Pt was purchased from Fluidigm. Indium chloride containing 95% ^115^In and 5% ^113^In was purchased from Trace Sciences. Following previously published methods (59), indium was conjugated to an anti-CD45 antibody (clone HI30, BioLegend).

Cryopreserved PBMCs and BALF cells were thawed at 37°C. After washing and centrifugation, samples were passed through a filter and collected (Falcon). After cells were counted using a Muse Cell Analyzer (BM Bio), approximately 1 × 10^6^ cells were applied to 96-well Falcon V-bottom plates. In this study, because relatively few frozen BALF cells were recovered, we could not perform CyTOF analysis using BALF cells. A total of 1 μM Cell-ID Cisplatin-^198^Pt (Fluigidm) was added for 5 minutes. Human Fc Receptor Blocking Reagent (Miltenyi Biotec) was added at a 1:25 dilution for 15 minutes, followed by barcoding of each sample with 6 types of staining patterns using 3 types of anti-CD45 antibodies. All barcoded samples were combined and stained with antibodies specific for surface markers, and then some were stained with secondary antibodies. After fixation with Fixation/Permeabilization Concentrate (Thermo Fisher Scientific), samples were stained with antibodies specific for intracellular markers in permeabilization buffer (Thermo Fisher Scientific) and then some were also stained with secondary antibodies. They were incubated overnight in 1 mL Maxpar Fix and Perm Buffer (Fluidigm) with 1 μM Cell-ID Intercalator-Ir (Fluidigm). The pooled sample consisting of 15% EQ Four Element Calibration Beads (Fluidigm) with Cell Staining Buffer (Fluidigm) was measured using a CyTOF Helios system (Fluidigm).

Normalization beads were used to analyze data, followed by doublet removal based on 191 Ir intensity and dead cell removal by 198 Cisplatin intensity. Filtered cells were then gated for CD45^+^ cells to identify immune cells. The above filtering process was performed using FlowJo 10.5.0. CyTOF marker intensities were compensated by CATALST 1.14.1 (60) with data of single-stained beads. All CyTOF data were combined and transformed using arcsinh with cofactor 5 by the cytofkit (1.11.3) “cytof_exprsMerge” function (61). All samples were assessed using principal component analysis, and we corrected for batch effects using 15 PCs by harmony 1.0 (62). Batch correction of data was performed dimensionally reduction by UMAP using the uwot 0.1.10 package and Shared Nearest Neighbor and Louvain clustering with the buildSNNGraph function from the scran 1.18.7 package (63) and the cluster_louvain function from the igraph 1.2.6 package. Cell types in clusters were annotated manually using the median marker intensity. The above data processing was performed using R 4.0.5 and Rstudio 1.1.442.

### RNA sequencing of BALF cells.

RNA was extracted from BALF cells stored in RNAlater. An miRNeasy Mini Kit (Qiagen) was utilized for total RNA extraction. Full-length cDNA was generated using a SMART-Seq HT Kit (Takara Bio). An Illumina library was prepared using a Nextera DNA Library Preparation Kit (Illumina) according to the SMARTer Kit instructions. Sequencing was performed on an Illumina NovaSeq 6000 sequencer (Illumina) in the 100-base single-end mode. Sequenced reads were mapped to the human reference genome sequence (hg19) using TopHat v2.0.12. Fragments per kilobase of exons per million mapped fragments were calculated using Cufflinks v2.1.1. Raw data were deposited in the NCBI’s Gene Expression Omnibus database (GEO GSE193049).

### Murine model and cell line studies.

Female C57BL/6J mice aged 7 weeks were purchased from Japan SLC. Mice were housed in laminar flow rooms at a constant temperature and humidity in a pathogen-free facility, with sufficient food and water.

For tumor studies, KP cells were obtained from E.A. Akbay (University of Texas Southwestern Medical Center, Dallas, Texas, USA) (16). KP cells expressing either OVA or CXCL9 were generated using a pMX retroviral vector system as previously described (64, 65). In brief, the full segment of OVA was amplified by PCR and cloned into a pMX retroviral vector at the BamHI and SalI restrictions sites. The mouse CXCL9 coding region (GenScript NM_008599.4) was synthesized with 5′ EcoRI and 3′ Not I restriction sites and then cloned into a pMX retroviral vector at the corresponding sites. Retroviral supernatants were generated by transfecting the retrovirus packaging vector and each pMX vector containing the gene of interest into the 293T cell line. After transduction with 8 μg/mL polybrene, single-cell derived clones were obtained by limiting dilution. The expression of OVA in KP cells was confirmed by IFN-γ production after coculture with the splenocytes from an OT-I mouse. The expression of mouse CXCL9 was also confirmed by ELISA of the transduced cell culture supernatant. All cell lines were maintained in complete RPMI 1640 medium (RPMI 1640 medium containing 10% heat-inactivated FBS [Gibco] and 1% penicillin–streptomycin [Nacalai Tesque]).

### Measurement of CXCL9 and H-2K^b^-SIINFEKL expression in KP^OVA-mock^ and KP^OVA-Cxcl9^ cell lines.

For measurement of CXCL9, cells were stimulated overnight with lipopolysaccharide 2 hours after IFN-γ priming in complete RPMI 1640 medium. After spinning and washing with 2% FBS in PBS, cells were subjected to surface staining with LIVE/DEAD Fixable Aqua Dead Cell Stain (Invitrogen). Then, cells were treated with fixation/permeabilization buffers (Thermo Fisher Scientific), and intracellular CXCL9 staining was performed. For measurement of H-2K^b^-SIINFEKL, cells were stimulated with 20 ng/mL IFN-γ in complete RPMI 1640 medium overnight. After surface staining with LIVE/DEAD Fixable Aqua Dead Cell Stain (Invitrogen), cells were stained with anti-mouse H-2K^b^ bound to SIINFEKL antibody (BioLegend).

### Cell proliferation assay.

To compare the proliferation rate of KP^OVA-mock^ and KP^OVA-Cxcl9^ cells, 2 × 10^3^ cells were seeded in 100 μL complete RPMI 1640 medium and cultured in 96-well flat-bottom plates. Then, the absorbance was measured at 450 nm using a microplate reader at baseline and 24 and 48 hours, according to Cell Counting Kit-8 protocol (DOJINDO).

### Murine lung tumor model and in vivo treatments.

All mice were anesthetized with subcutaneous administration of ketamine and xylazine. After skin incision, mice were then inoculated in the left lung with 1 × 10^6^ KP^OVA^ cells in 20 μL PBS mixed with 20 μL Matrigel (Corning). For ABx treatment, mice were treated with a cocktail of ampicillin (1 g/L), neomycin (1 g/L), vancomycin (0.5 g/L), and metronidazole (0.5 g/L), or with water sterilized by autoclave, starting 2 weeks before tumor injection and continuing throughout the experiment. Mice undergoing anti–PD-1 therapy were i.p. injected with anti-mouse PD-1 antibody (RMP1-14; BioXcell) for 2 weeks (3 times/week) starting 3 days after tumor injection. Control groups were injected with rat IgG2a isotype control (2A3; BioXcell) in the same manner.

### BAL procedure in mice.

After mice were sacrificed by CO_2_ inhalation in a CO_2_ chamber, a 24-gauge Surflo Catheter (TERUMO) was inserted into the trachea. Then, the stylet hub was removed, and the catheter and trachea were firmly tied together with a nylon string. A 1 mL syringe containing 500 μL PBS was used to inject and aspirate the PBS 2 times, and then the syringe was removed from the catheter.

### Murine BALF flow cytometry.

After centrifugation of collected BALF, BALF cells were subjected to red blood cell lysis buffer (Thermo Fisher Scientific). Next, BALF cells were treated with LIVE/DEAD Fixable Aqua Dead Cell Stain (Invitrogen). After washing, cells were treated with Fc block (BioLegend) and then subjected to surface staining (Supplemental Table 4). After washing, cell populations were acquired using the flow cytometer.

### Measurement of CXCL9 in murine tumors by ELISA.

Tumor nodules were enucleated, and their weights were measured. They were then homogenized with scissors to form a paste and diluted with PBS (50 μL/mg) in an Eppendorf tube. Next, the mixture was incubated using a microtube mixer for 2 hours at 4°C, and tumor cells were spun down by centrifugation and supernatant was collected. The CXCL9 concentration in the supernatant was measured using a Duoset ELISA Kit (R&D Systems).

### Measurement of CXCL9 in tumor-infiltrating lymphocytes and tumor cells.

Lung tumor nodules were shredded into small pieces with scissors and incubated in an enzyme solution containing 100 U/mL collagenase type IV (Thermo Fisher Scientific) and 100 μg/mL DNase I (MilliporeSigma) for 45 minutes at 37°C. Then, cells were treated with ACK Lysing Buffer (Thermo Fisher Scientific) for 5 minutes at 37°C and passed through a 100 μm cell strainer (Falcon). Cells were incubated overnight in complete RPMI 1640 with lipopolysaccharide stimulation 2 hours after IFN-γ priming. After centrifugation and washing with 2% FBS in PBS, cells were treated with LIVE/DEAD Fixable Aqua Dead Cell Stain (Invitrogen). After Fc blocking, cells were subjected to surface staining (Supplemental Table 4). Then, cells were treated with fixation/permeabilization buffers, and intracellular CXCL9 staining was performed.

### Statistics.

All statistical analyses except for microbiome and CyTOF analyses were performed using GraphPad Prism software (version 8.1.0). Numerical data are presented as mean ± SEM or mean ± SD. The Mann-Whitney *U* test or 2-tailed Student’s *t* test was used to compare 2 groups. One-way ANOVA with multiple comparisons was used to compare 3 or more groups. Correlation analyses were performed with Spearman’s correlation with 2-tailed significance. *P* values of less than 0.05 were considered significant. *r* refers to Spearman’s correlation coefficient.

### Study approval.

All patient samples were obtained from participants who provided informed consent for BALF and blood collection in accordance with the Declaration of Helsinki and with approval from the ethical review boards of the Osaka International Cancer Institute (1609239124) and Osaka Habikino Medical Center (772). This study was registered in the University Hospital Medical Information Network Clinical Trials Registry (Tokyo, Japan) (UMIN000023822). All research on human participants was performed in accordance with its protocol. All experiments followed the approved guidelines of the Institute of Experimental Animal Sciences at Osaka University Medical School.

## Author contributions

KM, KF, YN, T. Hirai, AO, and YA performed the experiments and analyzed the data. KM, MT, KF, SK, YN, AO, HS, NO, TS, KN, YT, TK, and T. Hirashima collected the clinical specimens and analyzed the clinical data. TN, YKK, A. Kajihara, TM, SI, SU, TI, DO, and TA provided technical support. MT, SK, T. Hirashima, and A. Kumanogoh conceived the project. All authors contributed to writing the paper and discussing the content. KM, MT, and KF contributed equally as co–first authors. KM is listed first because KM performed most of all experiments and the integrated analysis. MT was listed next because MT made the greatest contribution to collecting clinical samples and analyzing the clinical data. KF analyzed the microbiome samples from collected BALF.

## Supplementary Material

Supplemental data

## Figures and Tables

**Table 1:**
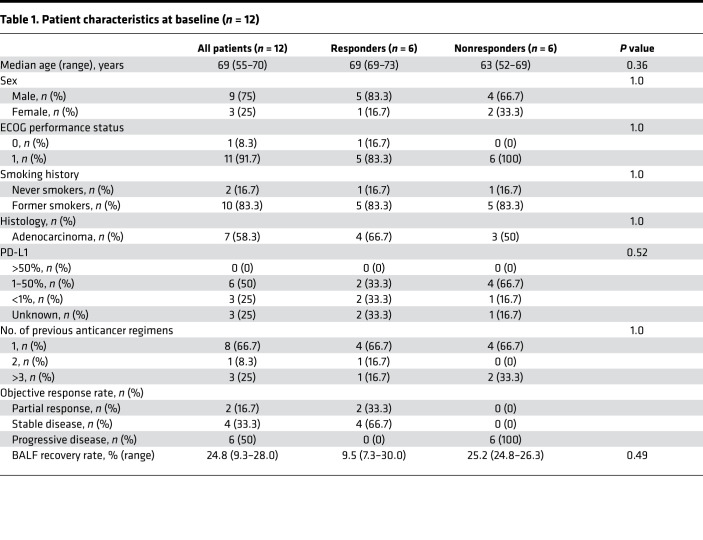
Patient characteristics at baseline (*n* = 12)

## Version 1 04/07/2022

In-Press Preview

## Version 2 05/09/2022

Electronic publication
