# Supplementary material for: Bronchoalveolar lavage fluid reveals factors contributing to the efficacy of PD-1 blockade in lung cancer
Source: JCI Insight. 2022 May 9;7(9):e157915. doi: 10.1172/jci.insight.157915 (PMC9090256; doi:10.1172/jci.insight.157915)
Supplement: Supplemental data [file jciinsight-7-157915-s289.pdf]

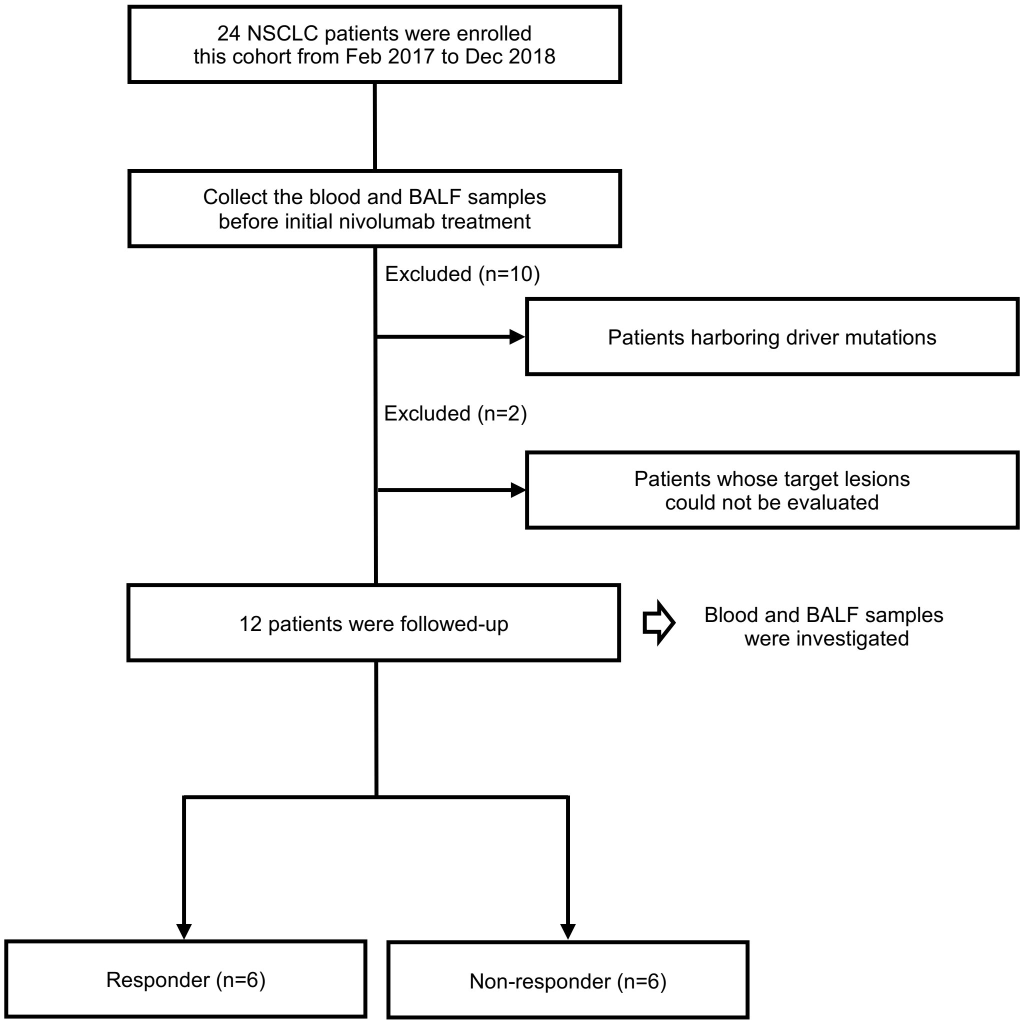

Supplemental Figure 1. Diagram of patient disposition.

Twenty-four NSCLC patients who started nivolumab treatment as second- or further-line treatment between February 2017 and December 2018 were enrolled in this cohort. Among these patients, 10 harboring driver mutations and 2 assessed as “not evaluable (NE)” were excluded. Finally, six patients with partial response (PR) or stable disease (SD) were assigned to the responder group, and the other six with progressive disease (PD) were assigned to the non-responder group.

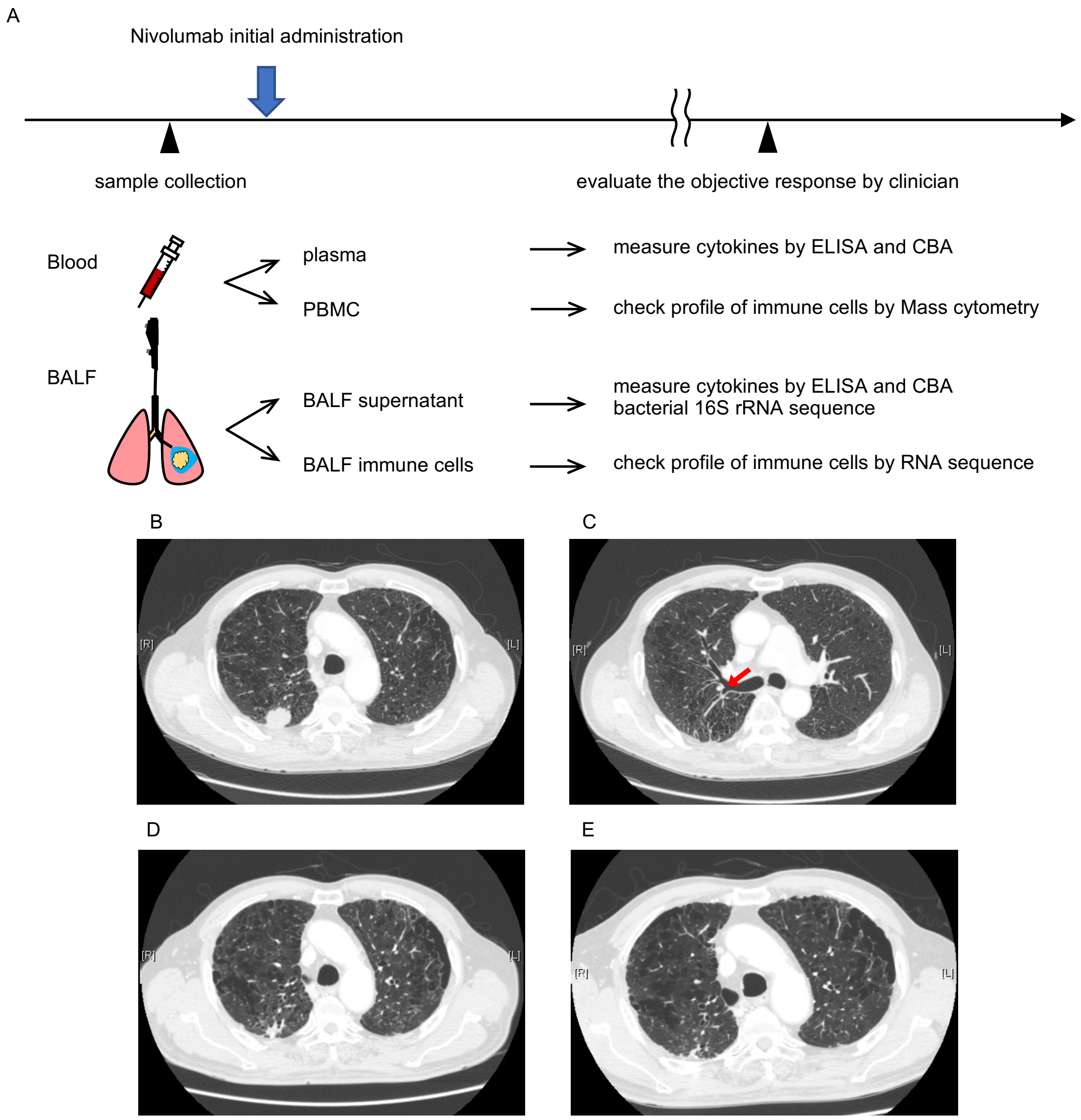

Supplemental Figure 2. Study protocol.

(**A**) BALF and blood samples were collected before initial nivolumab administration. Cells and supernatant were isolated and stored at −80°C. Immune profiles of BALF cells were analyzed by bulk RNA sequencing and blood cells were assessed using cytometry by time of flight (CyTOF). Cytokines in BALF supernatant and plasma were measured by ELISA and CBA. (**B−E**) Representative chest computed tomography images of a responder are shown. (**B**) The target tumor is located in the right upper lobe and is connected to right B^2a. (**C**) BAL was performed in right B^2a (red arrow). (**D**) Four months after nivolumab initiation. (**E**) Forty-five months after nivolumab initiation.

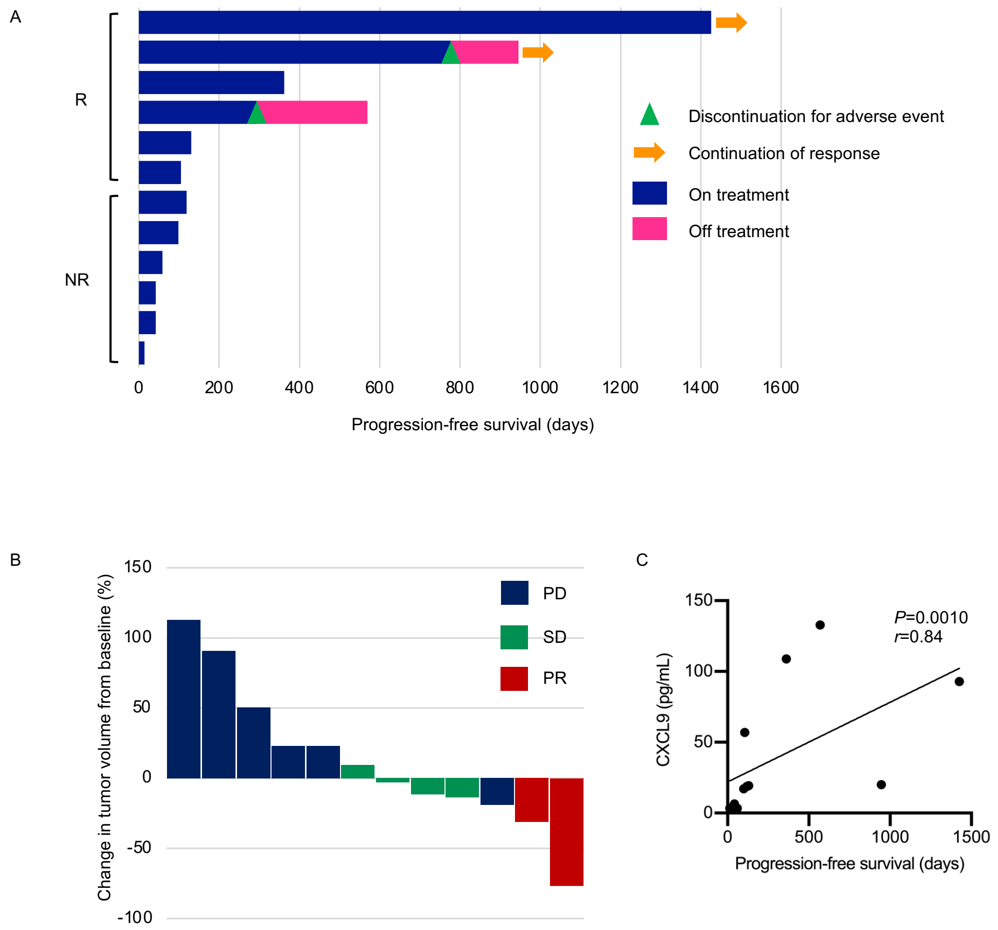

Supplemental Figure 3. Clinical outcomes.

(**A**) Swimmer plot of progression-free survival of the 12 patients evaluated in this study. R corresponds to responders and NR to non-responders. (**B**) Waterfall plot of percentage change in tumor volume from baseline. Tumor volumes were calculated based on the sum of the diameters of target lesions, including primary tumors and metastatic tumors, according to the RECIST criteria. Responders were defined as PR or SD, and non-responders as PD. Tumor volumes shrank in one non-responder (third from the right) but a new lesion appeared in the liver. (**C**) Correlation between CXCL9 levels in BALF and progression-free survival. Statistical analyses were performed using Spearman's correlation with two-tailed significance. *r*, Spearman's correlation coefficient.

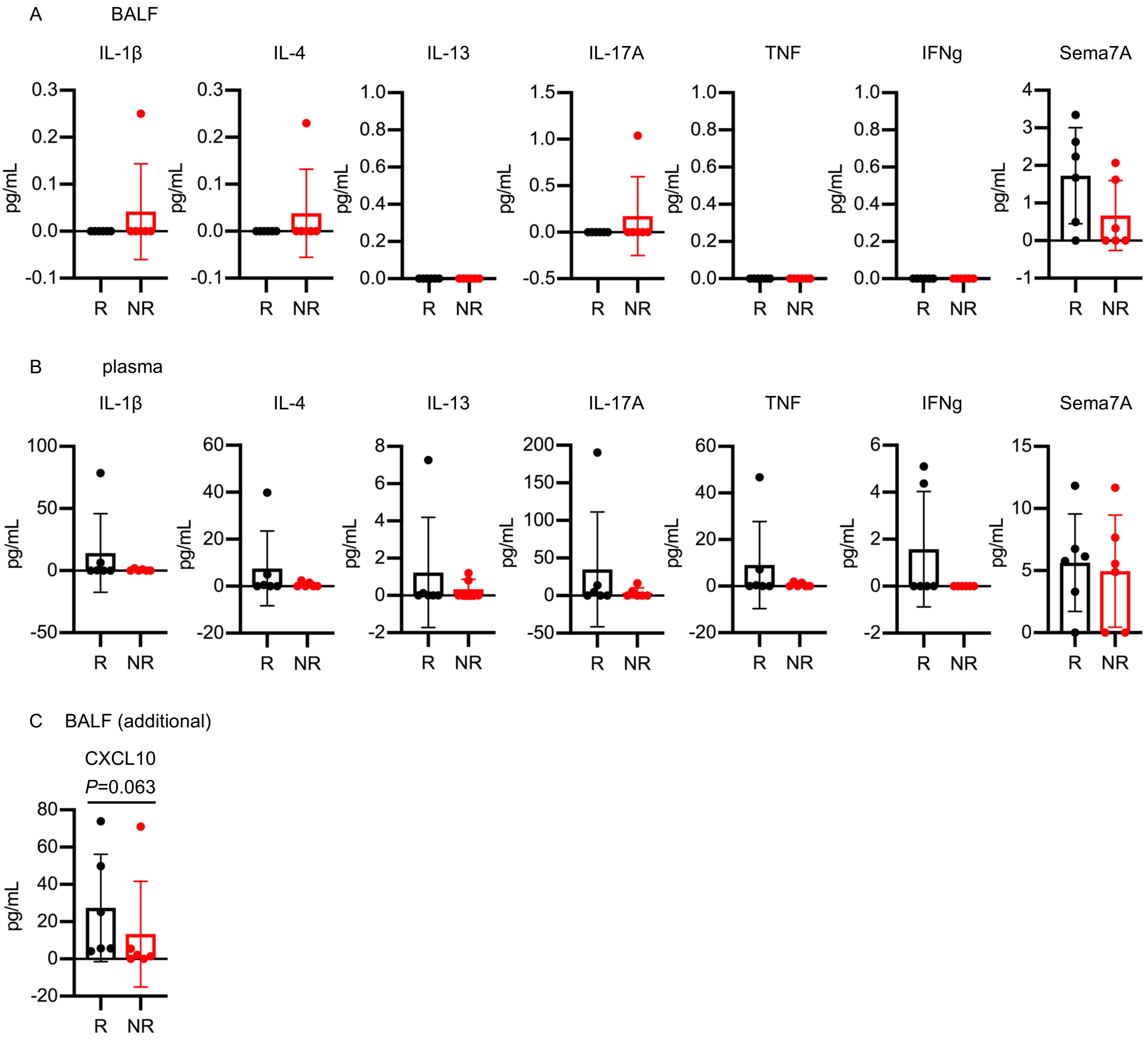

Supplemental Figure 4. Cytokine levels in BALF and plasma.

Cytokine levels in BALF (**A**) and plasma (**B**) were measured by ELISA or CBA and compared between responders (R: black) and non-responders (NR: red) before initial nivolumab treatment. (**C**) CXCL10 levels were measured after an additional freeze–thaw process. Data are presented as the mean ± SEM. There was no significant difference between the two groups. Statistical analyses were performed by the Mann-Whitney U test.

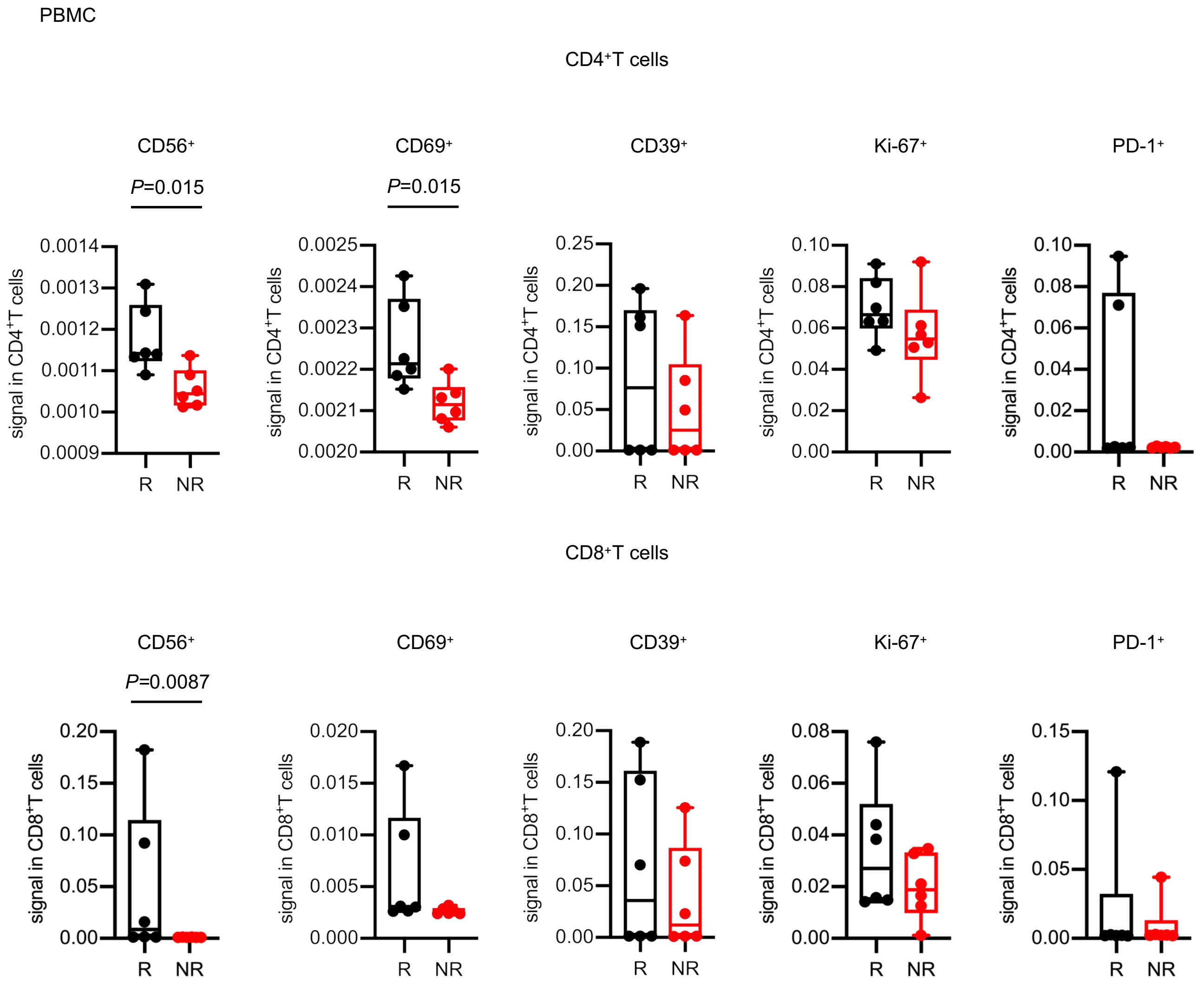

Supplemental Figure 5. Representative markers of CD4^{+} T cells and CD8^{+} T cells in PBMCs.

Comparison of immune cell profiles (the panel is shown in Supplemental Table 1) in peripheral blood mononuclear cells (PBMCs) between responders (R: black) and non-responders (NR: red) before initial nivolumab treatment. Profiles were assessed by CyTOF. The representative data shown indicate significant differences between the two groups. Data are presented as the mean ± SEM. Statistical significance was determined by the Mann-Whitney U test.

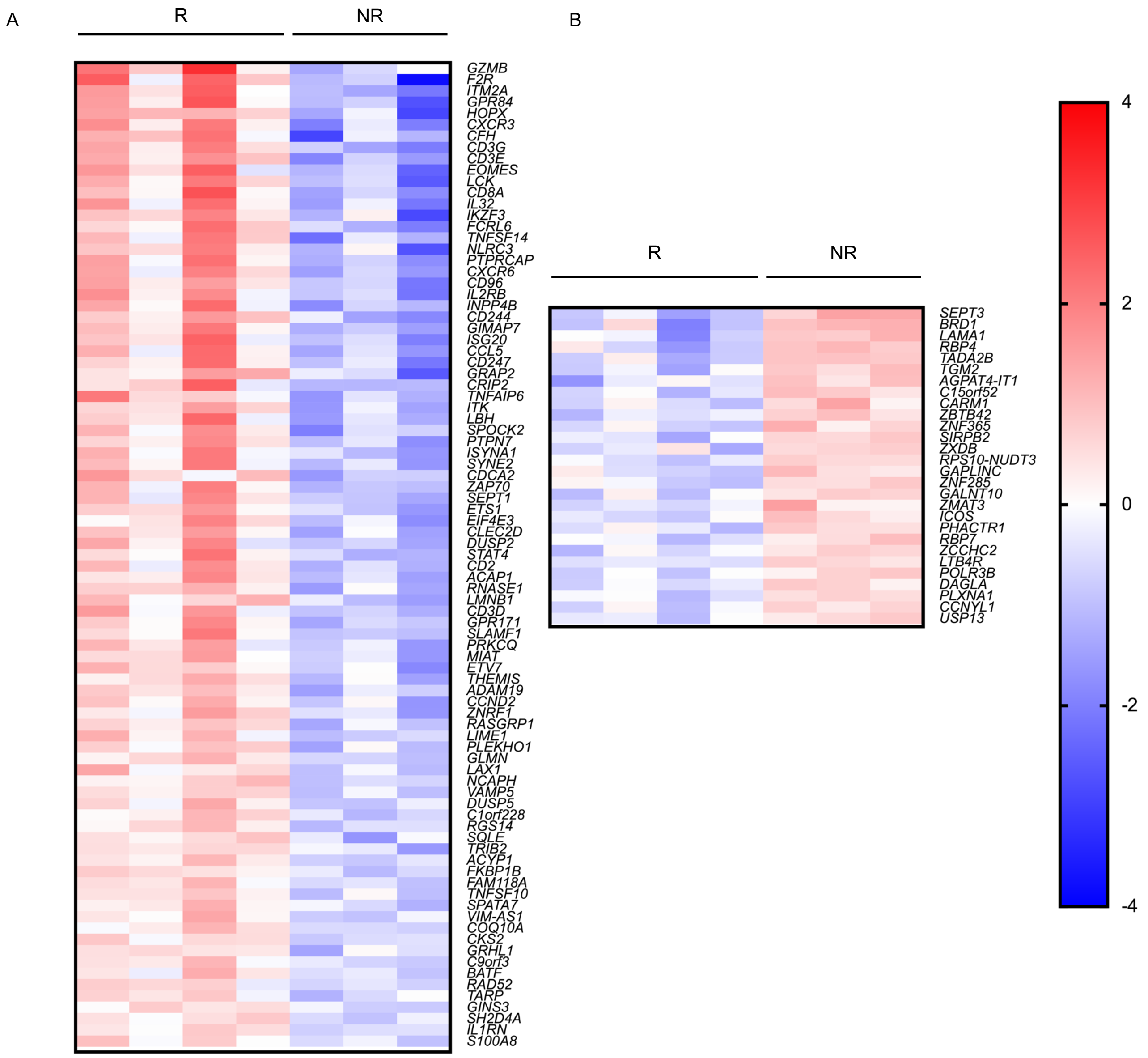

Supplemental Figure 6. Transcriptome profiling of BALF immune cells.

The transcriptome profiles of bulk cells collected from BALF were compared between four responders and three non-responders. One hundred fifteen representative genes (normalized counts >10) that were significantly (Student's t-test, $P < 0.05$) and differentially ($\log_2$ fold change >2) expressed in seven paired samples are shown as a heatmap. Eighty-seven genes were upregulated (**A**) and 28 were downregulated (**B**) in responders.

A

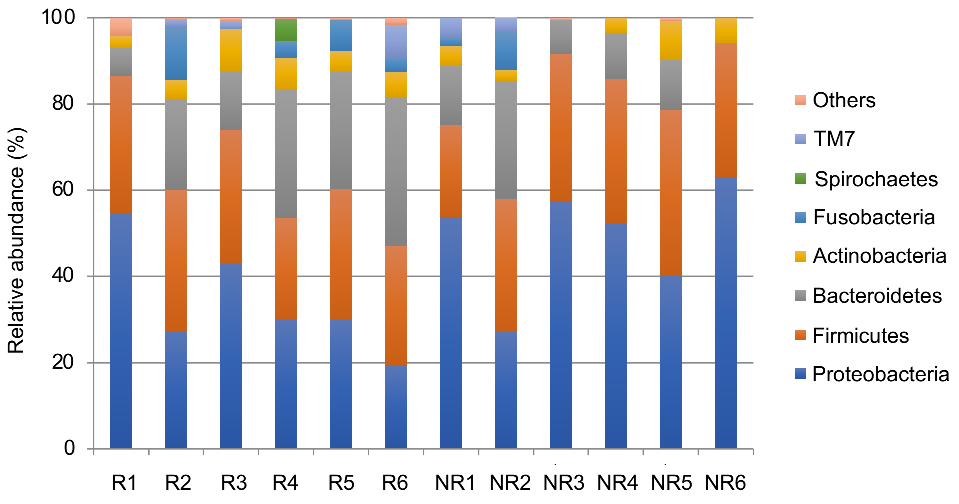

B

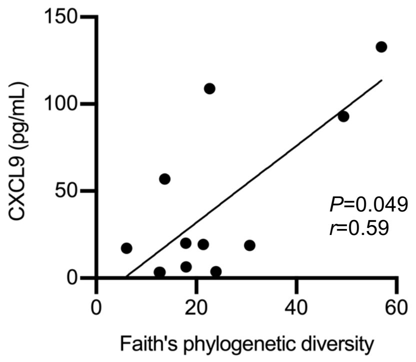

Supplemental Figure 7. Respiratory microbial composition of BALF samples and correlation between BALF CXCL9 levels and bacterial diversity.

(**A**) Respiratory microbial composition at the phylum level based on the relative abundance of operational taxonomic units (OTUs) for each BALF sample. R1–6 correspond to six responders and NR1–6 to six non-responders. (**B**) Correlation between CXCL9 levels and Faith's phylogenetic diversity in BALF. Statistical analyses were performed using Spearman's correlation with two-tailed significance. *r*, Spearman's correlation coefficient.

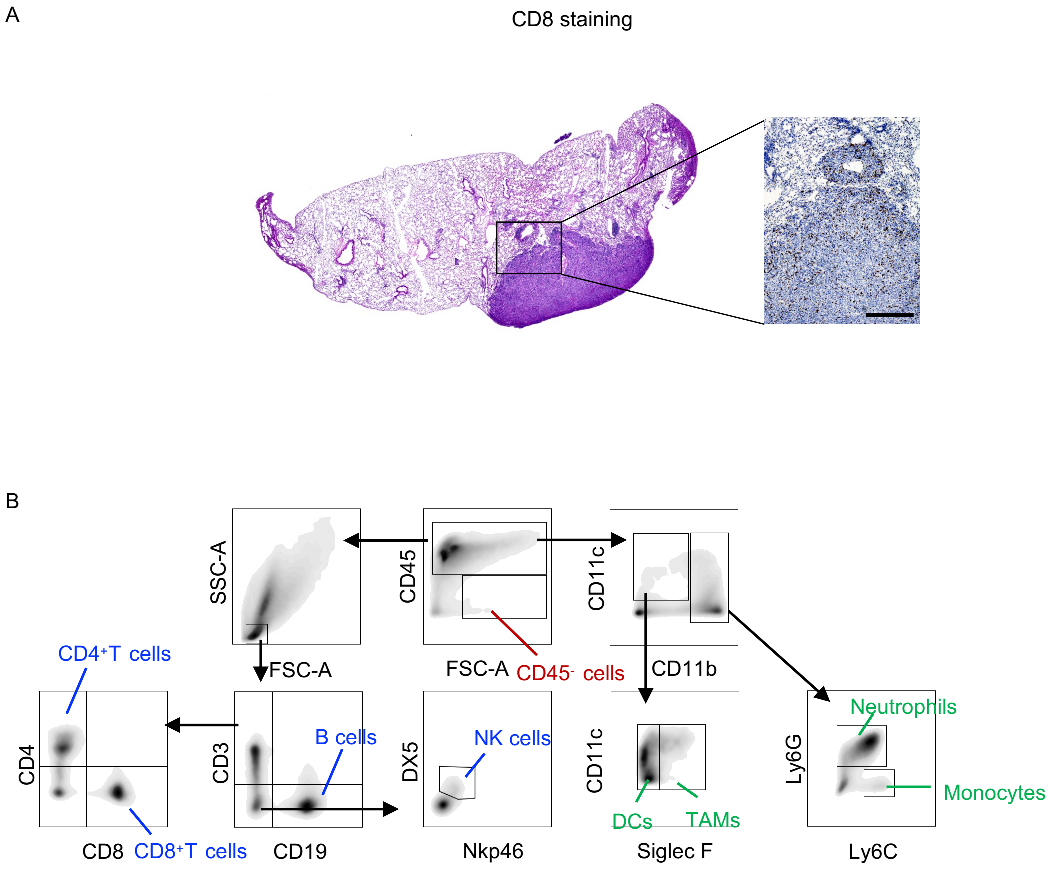

Supplemental Figure 8. Establishment of an intrathoracic model with KP^{OVA} cells.

KP^{OVA} cells were inoculated intrathoracically into the left lung of each mouse. Representative images are shown. (**A**) H&E staining of lung tissue 2 weeks after KP^{OVA} cell inoculation (left). Immunohistochemistry staining of CD8 (brown) in the tumor tissue. Magnification = 4x. Scale bar: 500 μm (right). (**B**) Gating strategy for flow cytometry analysis to identify which cell populations secrete CXCL9 in the tumor microenvironment.

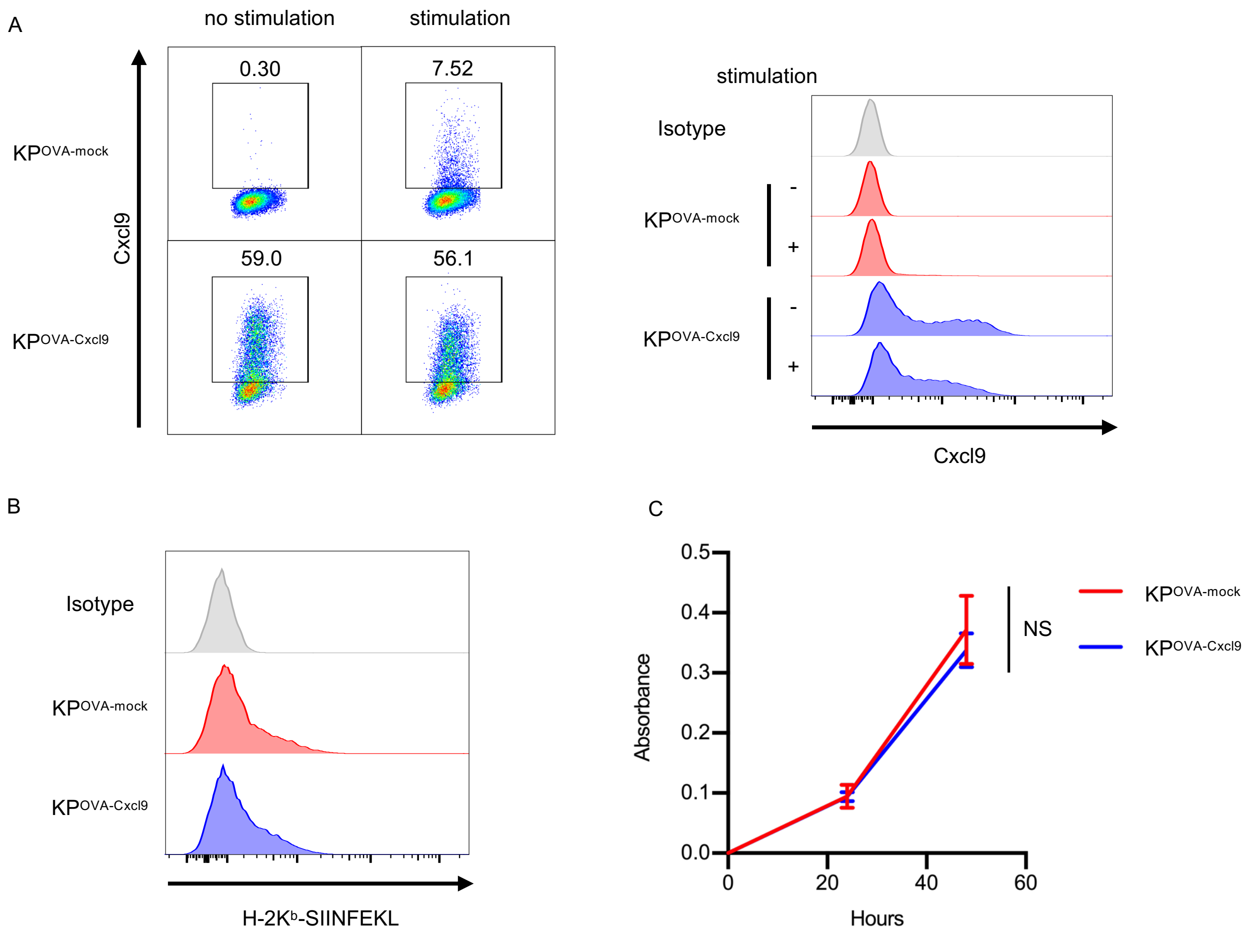

Supplemental Figure 9. Establishment of overexpression of CXCL9 in KP^{OVA} cells.

Flow cytometry analysis comparing KP$^{OVA\text{-}mock}$ and KP$^{OVA\text{-}Cxcl9}$ with or without stimulation (**A**) and with H-2K^{b}-SIINFEKL induction by IFN-γ (**B**). (**C**) Cell proliferation rates were compared between KP$^{OVA\text{-}mock}$ (red) and KP$^{OVA\text{-}Cxcl9}$ (blue) for 48 hours. Data are presented as the mean ± SD. Statistical significance determined by Student's t-test. NS, not significant (n = 6/group).

Supplemental Table 1. List of antibodies (human) used in CyTOF analysis.

Antibody	Clone	Manufacturer
Anti-Human CD45-Maxpar®Ready	HI30	BioLegend
Anti-Human CD19-142Nd	HIB19	Fluidigm
Anti-Human CD69-144Nd	FN50	Fluidigm
Anti-Human CD138-145Nd	DL-101	Fluidigm
Anti-Human CD8a-146Nd	RPA-T8	Fluidigm
Anti-Human CD11c-147Sm	Bu15	Fluidigm
Anti-Human CD16-Maxpar®Ready	3G8	BioLegend
Anti-Human CD25-149Sm	2A3	Fluidigm
Anti-Human CD86-150Nd	IT2.2	Fluidigm
Anti-Human CD39 (ENTPD1)	498403	R&D Systems
Anti-Human CD185 (CXCR5)-153Eu	RF8B2	Fluidigm
Anti-Human CD279 (PD-1)-155Gd	EH12.2H7	Fluidigm
Anti-Human CD183 (CXCR3)-156Gd	G025H7	Fluidigm
Anti-Human CD194 (CCR4)-158Gd	L291H4	Fluidigm
Anti-Human CD197 (CCR7)-159Tb	G043H7	Fluidigm
Anti-Human CD28-160Gd	28.2	Fluidigm
Anti-Human CD152 (CTLA-4)-161Dy	14D3	Fluidigm
Anti-Human CD122 (IL-2Rβ)	27302	R&D Systems
Anti-Human CD56-161Dy	NCAM16.2	Fluidigm
Anti-Human Sema7A	310829	R&D Systems
Anti-PE-165Ho	PE001	Fluidigm
Anti-Human Foxp3-PE	236A/E7	eBioscience
Anti-Human Sema4A	741509	R&D Systems
Anti-Human CD27-Maxpar®Ready	O323	BioLegend
Anti-Human Ki-67-168Er	Ki-67	Fluidigm
Anti-Human CD45RA-169Tm	HI100	Fluidigm
Anti-Human CD3-170Er	UCHT1	Fluidigm
Anti-Human CD141 (Thrombomodulin)	501733	R&D Systems
Anti-Human CD134 (OX40)	977974	R&D Systems
Anti-Human HLA-DR-173Yb	L243	Fluidigm
Anti-Human CD4-174Yb	SK3	Fluidigm
Anti-Rabbit-175Lu	polyclonal	Fluidigm
Anti-Sema6D	polyclonal	R&D systems
Anti-Human CD11b-209Bi	ICRF44	Fluidigm
FcR Blocking Reagent, human	–	Miltenyi Biotec

Supplemental Table 2. Significantly upregulated genes in BALF cells of responders compared to non-responders.

Gene name	Fold change ratio	*P* value		Gene name	Fold change ratio	*P* value
GZMB	13.82	0.032		*SPOCK2*	3.63	0.033
F2R	9.62	0.034		*PTPN7*	3.60	0.020
ITM2A	6.28	0.015		*ISYNA1*	3.54	0.049
GPR84	6.12	0.032		*SYNE2*	3.52	0.046
HOPX	6.10	0.012		*CDCA2*	3.51	0.015
CXCR3	5.78	0.020		*ZAP70*	3.49	0.031
CFH	5.68	0.034		*SEPT1*	3.47	0.029
CD3G	5.43	0.008		*ETS1*	3.41	0.013
CD3E	5.43	0.004		*EIF4E3*	3.38	0.038
EOMES	5.43	0.041		*CLEC2D*	3.36	0.024
LCK	5.26	0.022		*DUSP2*	3.34	0.048
CD8A	4.85	0.033		*STAT4*	3.33	0.037
IL32	4.79	0.045		*CD2*	3.30	0.031
IKZF3	4.76	0.045		*ACAP1*	3.28	0.023
FCRL6	4.64	0.020		*RNASE1*	3.24	0.018
TNFSF14	4.62	0.030		*LMNB1*	3.22	0.014
NLRC3	4.60	0.044		*CD3D*	3.21	0.043
PTPRCAP	4.58	0.026		*GPR171*	3.17	0.028
CXCR6	4.50	0.019		*SLAMF1*	3.09	0.037
CD96	4.40	0.012		*PRKCQ*	2.95	0.049
IL2RB	4.27	0.036		*MIAT*	2.94	0.028
INPP4B	4.27	0.038		*ETV7*	2.92	0.034
CD244	4.22	0.011		*THEMIS*	2.84	0.024
GIMAP7	4.22	0.014		*ADAM19*	2.81	0.009
ISG20	4.12	0.047		*CCND2*	2.72	0.048
CCL5	4.09	0.044		*ZNRF1*	2.72	0.045
CD247	3.99	0.043		*RASGRP1*	2.71	0.010
GRAP2	3.99	0.043		*LIME1*	2.67	0.024
CRIP2	3.89	0.041		*PLEKHO1*	2.66	0.031
TNFAIP6	3.84	0.027		*GLMN*	2.57	0.005
ITK	3.76	0.010		*LAX1*	2.44	0.037
LBH	3.76	0.047		*NCAPH*	2.44	0.008
VAMP5	2.41	0.007		*COQ10A*	2.16	0.016
DUSP5	2.34	0.036		*CKS2*	2.13	0.009
C1orf228	2.33	0.017		*GRHL1*	2.11	0.033
RGS14	2.33	0.013		*C9orf3*	2.11	0.022
SQLE	2.32	0.044		*BATF*	2.07	0.050
TRIB2	2.31	0.028		*RAD52*	2.06	0.022
ACYP1	2.25	0.008		*TARP*	2.06	0.048
FKBP1B	2.24	0.005		*GINS3*	2.04	0.011
FAM118A	2.23	0.019		*SH2D4A*	2.03	0.014
TNFSF10	2.23	0.024		*IL1RN*	2.03	0.005
SPATA7	2.20	0.036		*S100A8*	2.02	0.041
VIM-AS1	2.17	0.045				

Supplemental Table 3. Significantly downregulated genes in BALF cells of responders compared to non-responders.

Gene name	Fold change ratio	*P* value		Gene name	Fold change ratio	*P* value
USP13	2.00	0.021		*RPS10-NUDT3*	2.16	0.006
CCNYL1	2.01	0.032		*ZXDB*	2.22	0.045
PLXNA1	2.02	0.021		*SIRPB2*	2.32	0.022
DAGLA	2.02	0.010		*ZNF365*	2.37	0.021
POLR3B	2.03	0.033		*ZBTB42*	2.37	0.008
LTB4R	2.04	0.000		*CARM1*	2.38	0.036
ZCCHC2	2.04	0.035		*C15orf52*	2.48	0.013
RBP7	2.06	0.031		*AGPAT4-IT1*	2.58	0.038
PHACTR1	2.10	0.024		*TGM2*	2.63	0.022
ICOS	2.10	0.016		*TADA2B*	2.95	0.011
ZMAT3	2.11	0.041		*RBP4*	3.12	0.020
GALNT10	2.11	0.049		*LAMA1*	3.14	0.027
ZNF285	2.13	0.026		*BRD1*	3.81	0.029
GAPLINC	2.15	0.033		*SEPT3*	4.04	0.003

Supplemental Table 4. List of antibodies (mouse) used in flow cytometry analysis.

Antigen	Clone	Fluorescence	Manufacturer
CD3e	17A2<br>145-2C11	APC/Cyanine7<br>PE/Cyanine7	BioLegend
CD4	GK1.5<br>RM4-5	PE/Cyanine7<br>APC	BioLegend
CD8a	53-6.7<br>KT15	APC/Cyanine7<br>FITC	BioLegend<br>MBL
CD45	30-F11	APC/Cyanine7	BioLegend
CD11b	M1/70	PE/Cyanine7	BioLegend
CD11c	N418	Brilliant Violet 421	BioLegend
CD19	6D5	Brilliant Violet 421	BioLegend
Ly-6C	HK1.4	FITC	BioLegend
Ly-6G	1A8	PerCP/Cyanine5.5	BioLegend
CD49b (pan-NK cells)	DX5	Alexa Fluor® 488	BioLegend
CD335 (Nkp46)	29A1.4	PerCP/Cyanine5.5	BioLegend
Siglec-F	E50-2440	Alexa Fluor® 647	BD Biosciences
CD183 (CXCR3)<br>Isotype	CXCR3-173<br>HTK888	PerCP/Cyanine5.5	BioLegend
CD279 (PD-1)<br>Isotype	29F.1A12<br>RTK2758	APC	BioLegend
CD69<br>Isotype	H1.2F3<br>HTK888	Brilliant Violet 421	BioLegend
CXCL9 (MIG)<br>Isotype	MIG-2F5.5<br>HTK888	PE	BioLegend
T-Select H-2K^{b}OVA Tetramer-SIINFEKL<br>Isotype	–	PE	MBL
H-2K^b bound to SIINFEKL<br>Isotype	25-D1.16<br>MOPC-21	APC	BioLegend
